# Effects of Dlx2 overexpression on the genes associated with the maxillary process in the early mouse embryo

**DOI:** 10.3389/fgene.2023.1085263

**Published:** 2023-02-20

**Authors:** Jian Sun, Jianfei Zhang, Qian Bian, Xudong Wang

**Affiliations:** ^1^ Shanghai Key Laboratory of Stomatology, National Center for Stomatology, National Clinical Research Center for Oral Diseases, Department of Oral and Cranio-Maxillofacial Surgery, College of Stomatology, Shanghai Research Institute of Stomatology, Shanghai Ninth People’s Hospital, Shanghai Jiao Tong University School of Medicine, Shanghai Jiao Tong University, Shanghai, China; ^2^ Shanghai Institute of Precision Medicine, Shanghai, China

**Keywords:** Dlx2, bulk RNA-seq, maxillary process, craniofacial development, scRNA-Seq

## Abstract

The transcription factor Dlx2 plays an important role in craniomaxillofacial development. Overexpression or null mutations of Dlx2 can lead to craniomaxillofacial malformation in mice. However, the transcriptional regulatory effects of Dlx2 during craniomaxillofacial development remain to be elucidated. Using a mouse model that stably overexpresses Dlx2 in neural crest cells, we comprehensively characterized the effects of Dlx2 overexpression on the early development of maxillary processes in mice by conducting bulk RNA-Seq, scRNA-Seq and CUT&Tag analyses. Bulk RNA-Seq results showed that the overexpression of Dlx2 resulted in substantial transcriptome changes in E10.5 maxillary prominences, with genes involved in RNA metabolism and neuronal development most significantly affected. The scRNA-Seq analysis suggests that overexpression of Dlx2 did not change the differentiation trajectory of mesenchymal cells during this development process. Rather, it restricted cell proliferation and caused precocious differentiation, which may contribute to the defects in craniomaxillofacial development. Moreover, the CUT&Tag analysis using DLX2 antibody revealed enrichment of MNT and Runx2 motifs at the putative DLX2 binding sites, suggesting they may play critical roles in mediating the transcriptional regulatory effects of Dlx2. Together, these results provide important insights for understanding the transcriptional regulatory network of *Dlx2* during craniofacial development.

## Introduction

*Dlx2* (Distal-less homeobox 2) is a member of the Dlx family transcription factors that play critical roles in forebrain and craniofacial development. In mice, *Dlx2* is located on chromosome 2 at 42.65 cM (Tan and Testa, 2021). During embryonic development, *Dlx2* is expressed in the epithelial cells of the maxillary and mandibular processes, as well as the cranial neural crest cells (CNCC)-derived mesenchyme, indicative of its significant regulatory functions during the development of craniomaxillofacial tissues.

*Dlx2* has been shown to regulate several critical signaling pathways involved in development and differentiation. *Dlx2* is a transcription activator for Wnt1 and can activate the Wnt/β-Catenin signaling pathway (Zeng et al., 2020). It can also promote the expression of TARBP2 and thus further activates the JNK/AKT signaling pathway (Fang et al., 2020). The Dlx2/GLS1/Gln metabolic axis is an important regulator of the TGF-β/Wnt-induced snail-dependent epithelial-mesenchymal transition (Lee et al., 2016).

The regulation of skeletogenesis by *Dlx2* has been extensively demonstrated *in vitro*. An experiment in human bone marrow mesenchymal stem cells confirmed that overexpression of *Dlx2* can upregulate the expression of osterix, BSP, and MSX2 and elevate cellular alkaline phosphatase activity in the early stage of osteogenesis induction. It can also upregulate OCN expression at a later stage, thereby accelerating the mineralization of BMSC (Qu et al., 2014; Zeng et al., 2020). Studies in MC3T3-E1 cells have also reached the same conclusion, that *Dlx2* overexpression can upregulate osteogenic related genes, such as *Alp* and *Msx2* (Sun et al., 2015). *Dlx2* overexpression can also stimulate both OCN and ALP promoter activity, thereby enhancing osteogenic differentiation (Zhang et al., 2019). MMP13 is a major collagenase that degrades aggrecan and type II collagen in the late stage of chondrogenesis. Its promoter contains two *Dlx2*-response elements. *Dlx2* can inhibit the expression of MMP13 and reduce cartilage degradation by directly combining with these two elements (Zhang et al., 2018).

In addition to regulating osteogenesis and chondrogenesis, *Dlx2* also plays a critical regulatory role in neural development. Mice lacking DLX1 and DLX2 have a time-dependent block in striatal differentiation (Anderson et al., 1997b), showed no detectable cell migration from the subcortical telencephalon to the neocortex and also had few GABA-expressing cells in the neocortex (Anderson et al., 1997a). The transient overexpression of the transcription factors *Ascl1* and *Dlx2* in neural progenitor cells is sufficient to induce neuronal morphology, GABAergic gene expression and synaptic electrophysiological maturity (Barretto et al., 2020).

Recent advances in the development of transgenic mouse models have provided critical insights for understanding craniofacial development and malformations (Chai and Maxson, 2006). Previous studies have shown that *Dlx2* deletion and overexpression mutants exhibit craniofacial malformations. It has also been revealed that a null mutation of *Dlx2* may cause odontogenic cells to reprogram into chondrocytes and express *Sox9* (Thomas et al., 1997). In E13.5 mouse dental germ, overexpression of *Dlx2* can also increase the expression of *Sox9* (Dai et al., 2017). Hence, it is speculated that *Sox9* may be a downstream effector of *Dlx2*. In addition, in mouse E13.5 dental germ that exhibits overexpression of *Dlx2*, the expression levels of TGFβR1, TGFβR2, Smad4, and *Msx2* are upregulated. In the epithelium, *Msx2* is also upregulated and the expression of *Runx2*, an osteogenic and odontogenic marker, is downregulated in dental germ and alveolar bone (Dai et al., 2017). This indicates that the overexpression of *Dlx2* may interfere with the development of tooth and bone through its interaction with these genes. However, the complex gene regulatory network, downstream of *Dlx2*, has not yet been fully described.

In our earlier work, we constructed a mouse model that can overexpress *Dlx2* in cells derived from neural crest cells (Sun et al., 2022). Such a mouse model enables us to determine the transcriptional effects of *Dlx2* overexpression on the mouse maxilla. In the present study, by comparing the transcriptomes of the maxillary process in E10.5 *Dlx2*-overexpressing mice and wild-type mice, we showed that the effect of *Dlx2* overexpression on the development of the maxillary process began at the earliest stage of maxillary process development and the transcriptional effect changed over time. Single-cell RNA sequencing (scRNA-Seq) of the early maxillary process showed that *Dlx2* inhibited cell proliferation and promoted cell differentiation without changing the trajectory of differentiation. Moreover, cleavage under targets and tagmentation (CUT&Tag) analysis revealed the putative target genes that *Dlx2* may interact with. These comprehensive analyses provide important insights for understanding the regulatory roles of *Dlx2* during craniofacial development and pave the road for further functional dissection of the downstream regulatory network of *Dlx2*.

## Materials and methods

### Animals

We obtained *wnt1* ^*cre*^ mice from the Jackson laboratory. The *Rosa26* ^*CAG-LSL-Dlx2−3xFlag*^ mouse was constructed by the Shanghai Model Organisms Center, Inc. (Shanghai, China). To generate *wnt1* ^*cre*^ *; Rosa26* ^*Dlx2/-*^ mice, which could specifically overexpress *Dlx2* in neural crest cells, we mated *wnt1* ^*cre*^ mice with *Rosa26* ^*CAG-LSL-Dlx2−3xFlag*^ mice. Wildtype C57BL/6J mice were purchased from Shanghai Jihui Laboratory Animal Care Co. Ltd. (Shanghai, China). All mice were maintained under SPF conditions at the Animal Center of the Ninth People’s Hospital affiliated with Shanghai Jiao Tong University School of Medicine. The day of the appearance of a vaginal plug was defined as E0.5 in all timed pregnancies. Embryos at the E10.5 and E12.5 stages (12:00 h of the day when the vaginal plug was detected was counted as E0.5) and P0 pups were collected for subsequent experiments. All animal experiments were approved by the Animal Care and Usage Committee of the Ninth People’s Hospital affiliated to Shanghai Jiao Tong University School of medicine.

### Micro-computed tomographic (micro-CT) imaging and 3D reconstruction

Micro-CT was performed using a SkyScan 1176 (Bruker, Germany). Micro-CT images were acquired from P0 mice, with an x-ray source voltage of 45 kV and current of 550 µA. The data were collected at a resolution of 18 µm. Volume rendering in 3D was achieved using Mimics Medical 21.0 (Materialize). We evaluated micro-CT scans from three replicates per genotype. All landmarks were determined based on *Mouse Development* (Eds. J Rossant and P.L.Tam, 2002) and www.getahead.la.psu.edu.

All bones used in this study were manually segmented. Micro-CT scanning data were uploaded to Mimics as DICOM files. The background noise from these segmentations and bones outside the scope of this study were manually removed using Mimics’ editor tools. The remaining craniofacial bones were isolated and labeled using pre-scale thresholds that allowed only bones to be labeled. Reconstruction data were then rendered using Mimics’ 3D calculation tools and analysis tools were used for the measurements of isolated bones. The mean measurements of the maxillary bones were compared between the P0 wildtype and *Dlx2* overexpression groups.

### Statistical analysis

GraphPad Prism v.8 for Windows (GraphPad Software, La Jolla, CA, United States) was used for the statistical analysis. For all graphs, error bars represent standard deviations. Independent two-tailed Student’s *t*-tests were applied for comparisons between two groups. Differences were considered to be statistically significant at *p*-values < 0.05.

### Isolation of mouse maxillary processes

In E10.5 and E12.5, pregnant C57BL/6 females were euthanized using isoflurane and cervical dislocation. The embryos were removed from the uterus into cold PBS and transferred into a 6 cm Petri dish, using a disposable glass straw. For sequences library construction, complete maxillary processes were carefully dissected out from embryos using micro tweezers under a stereomicroscope.

### Bulk RNA-seq and data analysis

Total RNA was extracted using Trizol from freshly dissected E10.5 maxillary process tissues. Three independent RNA samples were prepared for each genotype (WT and *wnt1* ^*cre*^ *; Rosa26* ^*Dlx2/-*^). We used 2 μg total RNA as input material for the library preparations for each sample. Sequencing libraries were generated using the NEBNext^®^ UltraTM RNA Library Prep Kit for Illumina^®^ (#E7530L, NEB, United States) following the manufacturer’s recommendations. The libraries were sequenced on an Illumina HiSeq X ten platform and 150 bp paired-end reads were generated. Sequenced reads were mapped to the mm 10 genome using STAR aligner version 2.7.3a. Comparisons between the RNA-seq datasets were performed using the DESeq2 package in *R*. Enrichment analyses and visualization of functional profiles of differentially expressed genes (DEGs) were performed using the clusterProfiler package in *R*.

### ScRNA-seq and UMAP analysis

Fresh maxillary process tissues were conserved in the GEXSCOPE^®^ Tissue Preservation Solution (Singleron) until library preparation. The scRNA-Seq libraries were constructed in accordance with the Singleron GEXSCOPE™ protocol from the GEXSCOPE™ Single-Cell RNA Library Kit (Singleron Biotechnologies). Pools were sequenced on the Illumina HiSeq X to generate 150 bp paired-end reads. Unsupervised clustering of cell populations was performed using the tSNE and UMAP analysis from the Seurat R package.

### CUT&Tag analysis

After obtaining fresh cells from the maxillary processes of E12.5 wildtype mice, the CUT&Tag libraries were prepared using the Hyperactive *In-Situ* ChIP Library Prep Kit for Illumina (Vazyme Biotech, TD901) as previously described (Zuo et al., 2021). Approximately 50,000 cells were used per sample. The Anti-DLX2 antibody (Abcam, ab272902) was used as the primary antibody and goat anti-rabbit IgG (Vazyme, Ab206-10-AA) was used as the secondary antibody. All CUT&Tag libraries were sequenced on the Illumina Nova Seq 6000 platform at PE150 mode. Low-quality reads and adapters were trimmed by Trim Galore (v0.6.5). The clean reads were mapped to the mm 10 genome using bowtie2 (v2.4.2).

## Results

### Micro-CT reveals abnormal bone formation in *Wnt1* ^*cre*^; *Rosa26* ^*Dlx2/-*^ mouse

**FIGURE 1:**
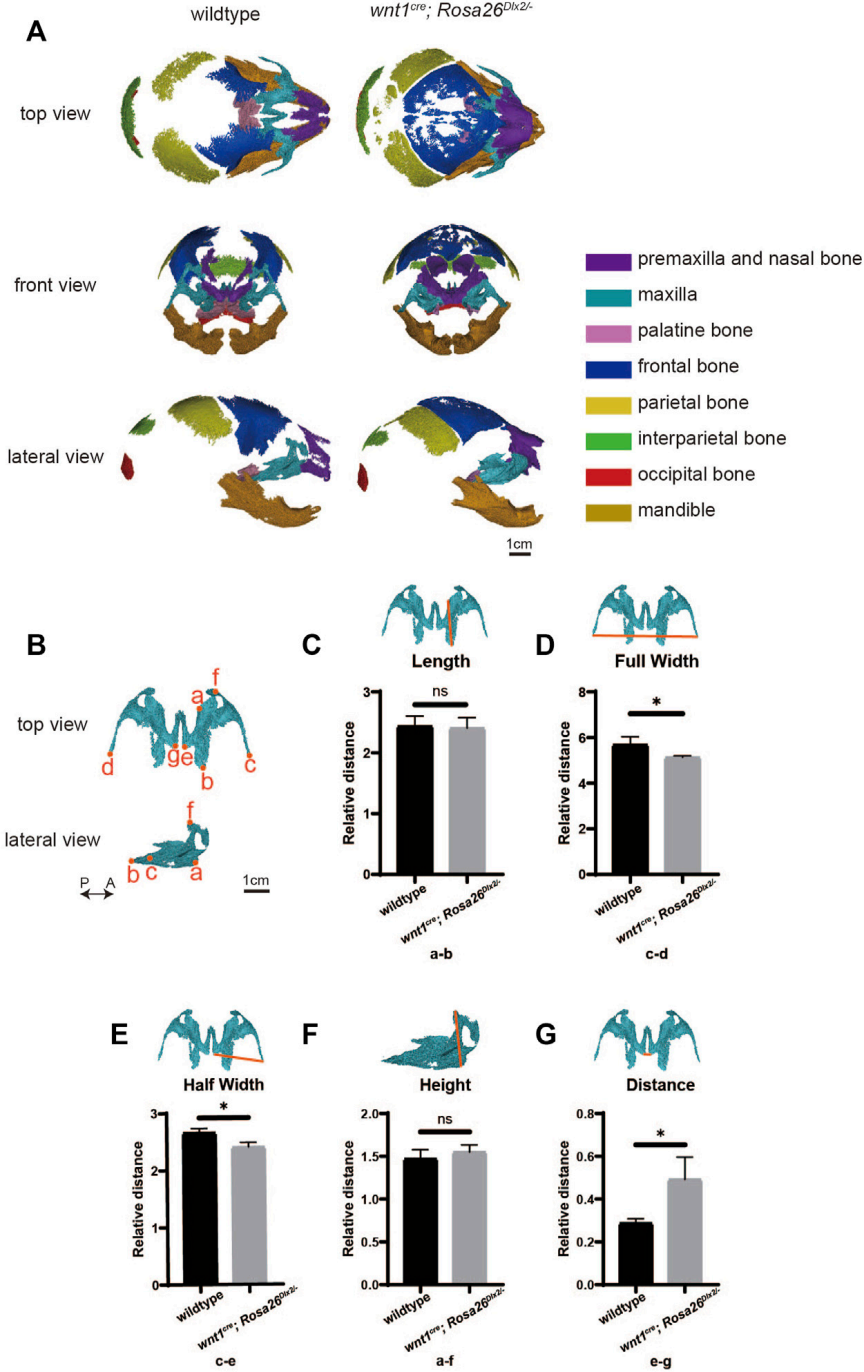
The conditional overexpression of *Dlx2* in cranial neural crest cells results in underdeveloped maxilla. **(A)** Top, front and lateral views of micro-computed tomographic rendering of a skull of a P0 wildtype and *wnt1* ^*cre*^; *Rosa26* ^*Dlx2/-*^ mouse. **(B)** Isolated maxilla from wildtype and *wnt1* ^*cre*^; *Rosa26* ^*Dlx2/-*^ mice. P←→A: Posterior to Anterior. Definitions of landmarks: a. Medial point of the premaxillary-maxillary suture; b. Posterior point of the maxilla; c. Right tip of the zygomatic process of maxilla; d. Left tip of the zygomatic process of maxilla; e. Right posterior-medial point of the palatine process of the maxilla; f. Anterior point of the maxilla; g. Left posterior-medial point of the palatine process of the maxilla. **(C–G)** Quantification of the size (length **(C)**, full width **(D)**, half width **(E)**, height **(F)**, and distance **(G)**) of the maxilla in wildtype and *wnt1* ^*cre*^; *Rosa26* ^*Dlx2/-*^ mice. *<0.05; ns. not significant. Definitions of landmarks: a. medial point of the premaxillary-maxillary suture; b. posterior point of the maxilla; cd. tip of the zygomatic process of maxilla; e.g., posterior-medial point of the palatine process of the maxilla; f. anterior point of the maxilla.

The *wnt1* ^*cre*^; *Rosa26* ^*Dlx2/-*^ mice, with *Dlx2* overexpressed in neural crest-derived cells exhibit craniofacial deformities such as cleft palate (Sun et al., 2022). To quantify the effect of *Dlx2* overexpression on craniofacial bone formation, we performed Micro-CT scanning on the head of P0 wild-type and *wnt1* ^*cre*^ *; Rosa26* ^*Dlx2/-*^ mice. Micro-CT analysis provides comprehensive information on anatomical landmarks and the size of each craniofacial bone (Ho et al., 2015). The premaxilla and nasal bone, maxilla, palatine bone, frontal bone, parietal bone, interparietal bone, occipital bone and mandible were isolated for analysis (Figure 1A). Obvious ectopic bone formation and absorption were found in the premaxilla and nasal bone, frontal bone and parietal bone of the *wnt1* ^*cre*^ *; Rosa26* ^*Dlx2/-*^ mice. The cranial anteroposterior diameter of the *wnt1* ^*cre*^ *; Rosa26* ^*Dlx2/-*^ mice was significantly smaller than that of wild-type mice. We isolated the maxilla from wild-type and *wnt1* ^*cre*^ *; Rosa26* ^*Dlx2/-*^ mice and defined the anatomical landmarks (Figure 1B). We next quantitatively compared the sizes of the maxilla using the landmarks (Figures 1C–G). The full width and half-width of the maxilla of the *wnt1* ^*cre*^ *; Rosa26* ^*Dlx2/-*^ mice were significantly decreased (Figures 1D, E) but there was no significant difference in the length and height (Figures 1C–F). The distance between the left and right halves of the maxilla in *wnt1* ^*cre*^ *; Rosa26* ^*Dlx2/-*^ mice was significantly increased (Figure 1G), which was consistent with the cleft palate phenotype. In summary, the quantitative comparison of Micro-CT scans revealed that *Dlx2* overexpression had a teratogenic effect on the mouse maxilla.

### *Dlx2* overexpression causes substantial gene expression changes in the E10.5 maxillary process

**FIGURE 2:**
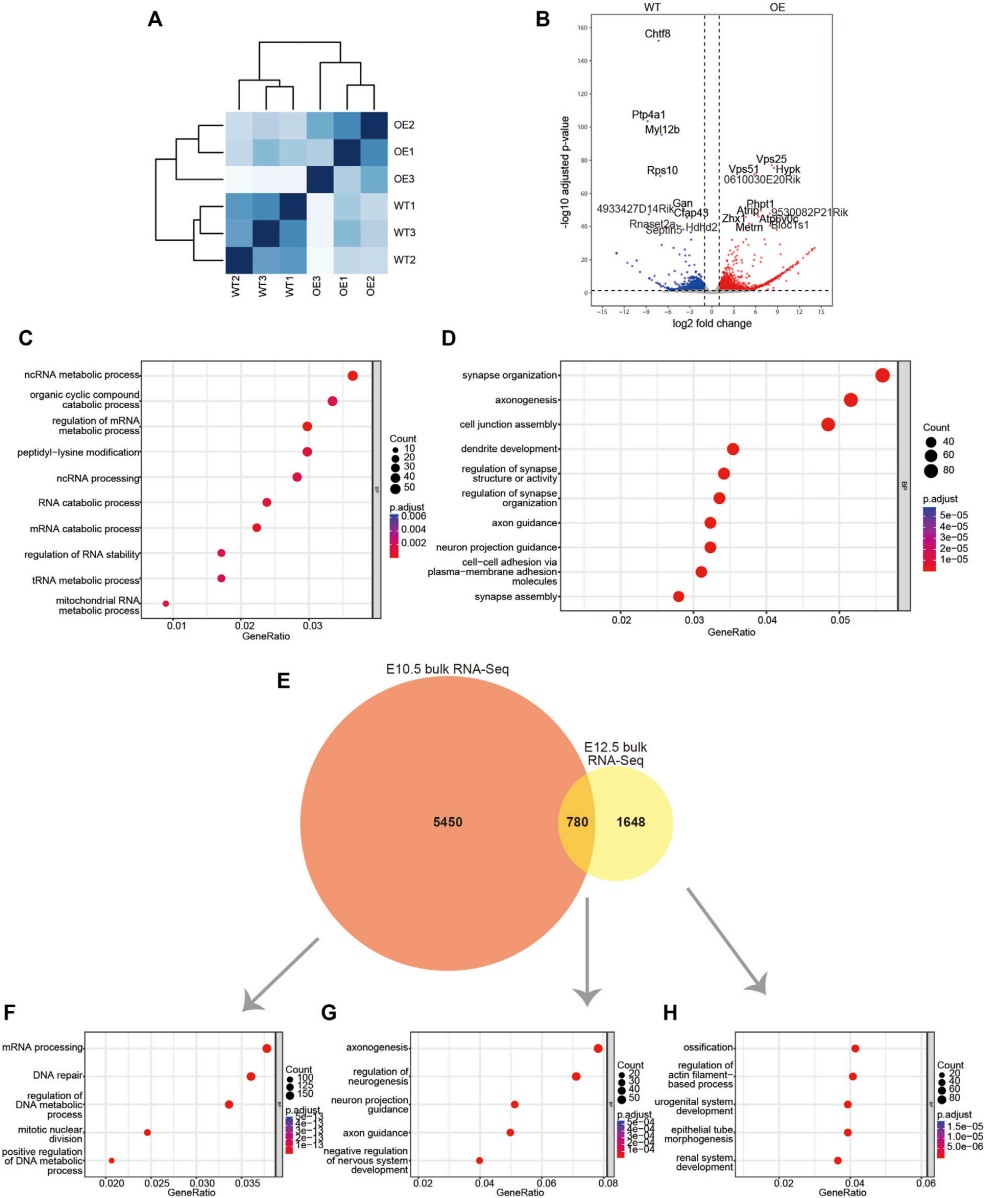
Bulk RNA-Seq data for the maxillary processes from E10.5 wildtype and *wnt1* ^*cre*^ *; Rosa26* ^*Dlx2/-*^ embryos. **(A)** Sample distances matrix showing the correlation between RNA-seq replicates. **(B)** Volcano plots to show differentially expressed genes between wildtype and *wnt1* ^*cre*^ *; Rosa26* ^*Dlx2/-*^ samples. **(C,D)** GO enrichment analysis of genes significantly upregulated or downregulated in *wnt1* ^*cre*^ *; Rosa26* ^*Dlx2/-*^. **(E)** Venn diagram to show the overlap between DEGs obtained from bulk RNA-Seq analysis of E10.5 and E12.5 mice. **(F–H)** GO enrichment analysis of genes expressed in E10.5 mice only **(F)**, E10.5-E12.5 overlap **(G)**, E12.5 only **(H)**. WT, wildtype; OE, *wnt1* ^*cre*^ *; Rosa26* ^*Dlx2/-*^.

In previous research, it was found that the overexpression of *Dlx2* had an impact on gene expression in E12.5 maxillary processes. However, the temporal and spatial expression analysis of *Dlx2* showed that the overexpression was already evident in the earliest stage (E10.5) of maxillary process formation (Sun et al., 2022). In order to further understand how the overexpression of *Dlx2* affects the development of maxillary processes, bulk RNA-Seq was performed on the maxillary processes of E10.5 wild-type and *wnt1* ^*cre*^ *; Rosa26* ^*Dlx2/-*^ mice. The individual replicates exhibited a high degree of correlation among the same genotype but a lower correlation was observed between replicates of different genotypes (Figure 2A), suggesting the overexpression of *Dlx2* already induced transcriptome changes at this stage.

Comparisons between the *wnt1* ^*cre*^ *; Rosa26* ^*Dlx2/-*^ and wild-type mice revealed that 6,230 genes exhibited significant expression changes. Of these genes, 2,192 genes were significantly upregulated and 1,762 genes were significantly downregulated (Figure 2B). The Gene Ontology (GO) enrichment analysis of DEGs that were significantly upregulated revealed that they are involved in critical biological processes and molecular pathways, such as organic cyclic compound catabolic process, peptidyl-lysine modification, ncRNA processing and RNA catabolic process. The upregulated genes were also involved in a variety of RNA metabolic processes, which included ncRNA metabolic process, regulation of mRNA metabolic process, tRNA metabolic process and mitochondrial RNA metabolic process (Figure 2C). Notably, the downregulated DEGs are enriched for functional terms related to neuronal development, such as synapse organization, axonogenesis, dendrite development and axon guidance (Figure 2D), which reflects the neural crest-origin of the maxillary processes.

We found the DEGs of E10.5 are significantly different from the previously reported bulk RNA-Seq DEGs of the mouse maxillary process in E12.5 wildtype and *wnt1* ^*cre*^ *; Rosa26* ^*Dlx2/-*^ mice (Sun et al., 2022). Between the 6230 E10.5 DEGs and the 2428 E12.5 DEGs, only 780 genes are common (Figure 2E). Among the 5,450 genes that were unique to E10.5, the most enriched GO terms are related to cell proliferation and transcription, such as mRNA processing, DNA repair, regulation of DNA metadata process and mitotic nuclear division (Figure 2F). The 780 DEGs that were shared by E10.5 and E12.5 are enriched for GO terms involved in neuronal development, such as axonogenesis, regulation of neurogenesis, neuron project guidance and axon guidance (Figure 2G). Notably, the 1648 DEGs that were unique to E12.5 mice are enriched for ossification related genes (Figure 2H). Thus, the overexpression of *Dlx2* can lead to different transcriptional responses and physiological outcomes at different stages of craniofacial development.

### *Dlx2* overexpression inhibits proliferation and promotes cell differentiation in maxillary process cells

Our bulk RNA-Seq analyses reveal pronounced transcriptional regulatory effects of *Dlx2* during early maxillary development. However, the inability to distinguish among different cell subpopulations within maxillary processes precludes further dissection of transcriptome changes associated with the differentiation of mesenchyme. Overcoming these limitations requires transcriptome profiling at single-cell resolution.

**FIGURE 3:**
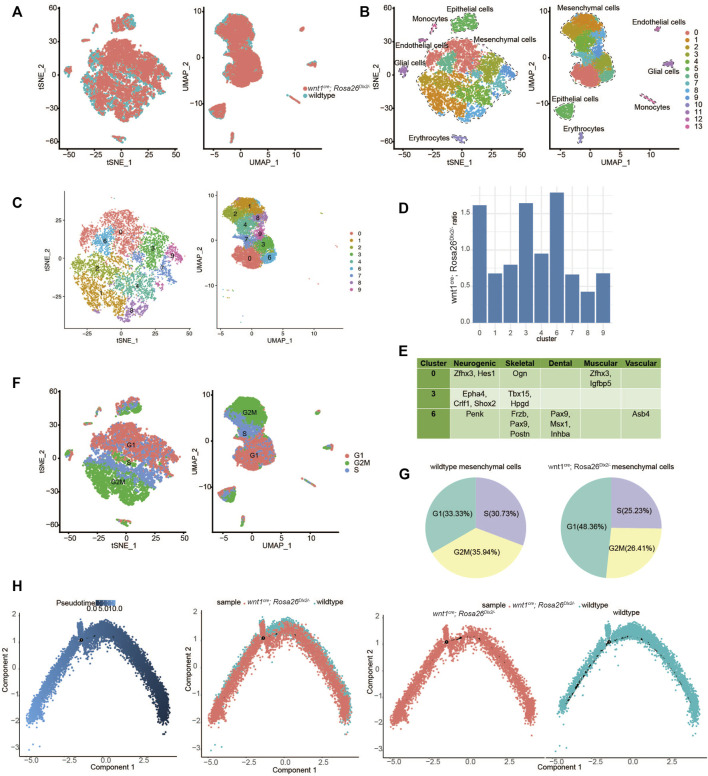
ScRNA-Seq analysis suggested that *Dlx2* overexpression inhibits proliferation and promotes cell differentiation of maxillary process cells. **(A)** Combined scRNA-Seq data of E12.5 wildtype and *wnt1* ^*cre*^; *Rosa26* ^*Dlx2/-*^ mice. The coincidence degree was high. **(B)** TSNE and UMAP showing all cell clustering of combined data. **(C)** TSNE and UMAP showing mesenchymal cell clustering of combined data. **(D)** The cell proportions of *wnt1* ^*cre*^ *; Rosa26* ^*Dlx2/-*^ mice samples to wildtype cells in each mesenchymal cell cluster corresponds to Figure 3C. It was higher in clusters 0, 3, 6, and lower in clusters 1, 8. **(E)** Some differentially expressed markers in clusters 0, 3, 6, which include multiple developmental systems. **(F)** Schematic diagram of cell cycle of mesenchymal cells after data was combined. **(G)** Pie chart of cell cycle proportion of wildtype (left) and *wnt1* ^*cre*^ *; Rosa26* ^*Dlx2/-*^ (right) mesenchymal cells in the combined data. **(H)** Pseudotime differentiation trajectories of combined data from E12.5 wildtype and *wnt1* ^*cre*^ *; Rosa26* ^*Dlx2/-*^ mice.

In the maxillary process at E10.5, the differentiation of most tissue types has not occurred and the mesenchymal cell population is relatively homogeneous. In order to more clearly reveal whether overexpression of *Dlx2* affects the differentiation trajectory of cells, we isolated the maxillary process tissues of E12.5 wild-type and *wnt1* ^*cre*^ *; Rosa26* ^*Dlx2/-*^ mice for single-cell RNA sequencing. The two scRNA-Seq datasets were combined and further analyzed. After dimensional reduction, the main cell populations from the two different samples largely overlapped with each other, indicating the batch effect was minimal (Figure 3A). The combined data were further clustered into 14 cell populations, with the largest cell populations corresponding to mesenchymal cells (Figure 3B). The nine mesenchymal clusters (clusters 0, 1, 2, 3, 4, 6, 7, 8, and 9) were selected for further analysis (Figure 3C).

In order to identify the cell populations most affected by *Dlx2* overexpression, we quantified the relative proportions of *wnt1* ^*cre*^ *; Rosa26* ^*Dlx2/-*^ mice samples to wild-type cells in each of the 9 mesenchymal cell clusters. In each cell cluster, the number of cells from *wnt1* ^*cre*^ *; Rosa26* ^*Dlx2/-*^ mice samples was divided by the number of cells from the wild-type samples. We found that the *wnt1* ^*cre*^ *; Rosa26* ^*Dlx2/-*^ cells were relatively enriched in clusters 0, 3, and 6, while depleted in the other clusters, particularly for clusters 1 and 8 (Figure 3D). To further understand the identities of clusters 0, 3, and 6, we examined their marker genes. For each of these clusters, several marker genes related to different tissue types can be found (Figure 3E), suggesting these cells may represent various precursor cells that have not fully committed to a specific cell type.

Notably, we also found that there are a large number of marker genes related to the cell cycle in each cell population (Supplementary Figure S1). We assessed the cell cycle stages for each mesenchymal cell and found that clusters 0, 3, and 6 mainly consisted of cells in the G1 phase, while clusters 1 and 8 consisted of cells in the G2M phase (Figure 3F). When comparing the cell cycle composition of mesenchymal cells from the two genotypes, we found that the proportion of cells in the G1 phase markedly increased, while the proportion of cells in G2M and S phase decreased in the *wnt1* ^*cre*^ *; Rosa26* ^*Dlx2/-*^ mice cells (Figure 3G). These results suggest the overexpression of *Dlx2* led to a slowdown of cell cycle progression and inhibition of cell proliferation.

To further understand how the overexpression of *Dlx2* affects the differentiation trajectory of maxillary mesenchymal cells, we performed pseudotime developmental trajectory analysis on the combined scRNA-Seq dataset. While the cells from wild-type and *Dlx2*-overexpressing mice exhibit similar trajectories without obvious divergence, the cells from the *wnt1* ^*cre*^ *; Rosa26* ^*Dlx2/-*^ mice were located at more downstream positions on the pseudotime trajectory compared to the wild-type cells (Figure 3H). This difference was further confirmed by quantifying the pseudotime scores for the cells from the two genotypes (Supplementary Figure S2). These analyses thus suggest the overexpression of *Dlx2* caused the mesenchymal cells within the maxillary process to enter a more differentiated state.

Taken together, our scRNA-Seq result suggests that overexpression of *Dlx2* had two related effects: inhibition of cell proliferation and promotion of differentiation. In *Dlx2*-overexpressing mice, the maxillary process cells may have precociously entered a more downstream differentiation state before they had sufficient proliferation, thereby impairing the development of the maxillary bone and ultimately causing the phenotypes of narrowing width, widening distance and cleft palate.

### CUT&Tag identifies candidate targets of DLX2

**FIGURE 4:**
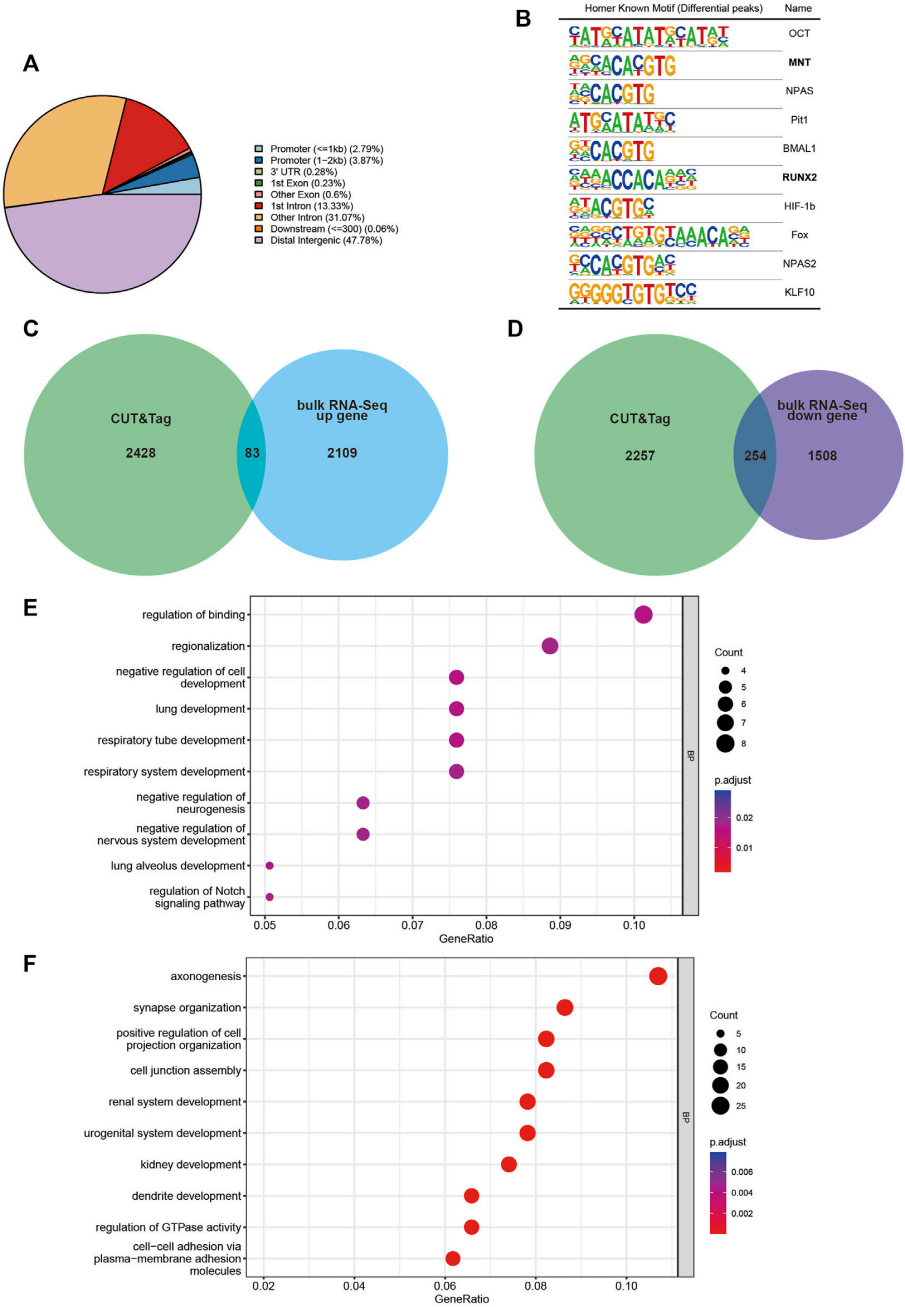
CUT&Tag analysis display of *Dlx2* downstream regulatory gene locus information. **(A)** Location of DLX2-occupancy peaks relative to the nearest annotated gene identified by CUT&Tag analysis. **(B)** Ten most enriched sequence motifs at DLX2-occupancy sites as determined using HOMER. The matched Motifs contained Mnt and Runx2. **(C,D)** Venn diagram to show the overlap between annotated genes identified by CUT&Tag analysis and E10.5 upregulated **(C)** or downregulated **(D)** DEGs. **(E, F)** GO enrichment analysis of the overlapping genes between annotated genes identified by CUT&Tag analysis and E10.5 upregulated **(E)** or downregulated **(F)** DEGs.

To uncover the regulatory mechanism of *Dlx2* in early maxillary process development, CUT&Tag analysis was performed. CUT&Tag is a novel and highly sensitive method used to identify transcription factor occupancy sites (Kaya-Okur et al., 2019; Kaya-Okur et al., 2020). We used this method to identify candidate direct targets of DLX2. We performed DLX2 CUT&Tag on two replicates of wild-type mice maxillary processes and identified 14,738 and 6899 peaks in each replicate. Intersection was used to obtain 3518 peaks that were common to both replicates. Through annotation of these peaks, we found that less than 7% were located in the promoter area (within 2 kb from the TSS) (Figure 4A). The largest proportion of DLX2 occupancy sites was located between genes, which indicated that DLX2 may bind to potential enhancer regions to regulate the expression of protein-coding genes (Figure 4A).

The ten most enriched known motifs identified by HOMER software are listed in Figure 4B. Among these enriched motifs, Runx2 was of particular interest because substantial *in vivo* and *in vitro* studies have shown that this gene is strongly associated with osteogenesis (Tosa et al., 2019; Deiana et al., 2020). This suggested that Dlx2 may function in collaboration with Runx2 to reshape the transcriptome when Dlx2 is overexpressed. Mnt is likely to be a transcriptional repressor and an antagonist of Myc-dependent transcriptional activation and cell growth (Hurlin et al., 1997), which may explain in part the inhibition of cell proliferation found by scRNA-Seq.

The CUT&Tag peak annotation identified 2,511 genes that are associated with DLX2 peaks. By cross-referencing these genes with bulk RNA-Seq upregulated DEGs, 83 upregulated *Dlx2* target genes were obtained (Figure 4C). These genes were enriched for genes involved in the regulation of binding, regionalization, negative regulation of cell development and regulation of Notch signaling pathway (Figure 4E). Interestingly, the Notch signaling pathway has been shown to play an important role in palatal development (Casey et al., 2006). The 254 downregulated *Dlx2* target genes were enriched for genes involved in axonogenesis and synapse organization (Figures 4D, F), consistent with the earlier analysis results. Among these downregulated genes, key osteogenic genes such as *Zeb2* (Wang et al., 2022) and *Rora* (Tao C et al., 2022) were significantly expressed in mesenchymal cell clusters 0 and 3 of scRNA-seq respectively, and these two clusters of cells constituted the majority of the mesenchymal cell group of *wnt1* ^*cre*^ *; Rosa26* ^*Dlx2/-*^. Overexpression of Dlx2 affects the osteogenesis of most mesenchymal cells. These putative *Dlx2* target genes may be the most direct effectors in the downstream regulatory network of *Dlx2*.

## Discussion

The conditional overexpression mouse model makes it possible to obtain stable *Dlx2* overexpression in mouse craniofacial tissues across different developmental stages. In previous work, we have performed a preliminarily exploration of the phenotypic characteristics of this mouse and described the gene expression changes of *Dlx2* overexpression in the maxillary process of E12.5 mice. The maxillary process is formed at the E9.5 stage. As *Dlx2* was overexpressed at the beginning of the maxillary process formation, we chose the earlier E10.5 maxillary process to describe the changes in gene expression. The DEGs at E10.5 share some similarities with those at E12.5, but there were also notable differences. Both sets of DEGs contain genes involved in the development of the nervous system, such as axonogenesis and regulation of neurogenesis. However, the DEGs specific to E10.5 are enriched for genes involved in RNA metabolism. In contrast, the DEGs specific to E12.5 are more enriched for genes involved in ossification. Such stage-dependent transcription effects may be attributed to several reasons. First, this may reflect the differences between the endogenous maxillary transcriptome at E10.5 *versus* E12.5, as early neurogenesis starts at E10.5 (Yun et al., 2002), which is slightly earlier than bone development. Second, maxillary cells at E10.5 and E12.5 may exhibit different chromatin accessibility landscapes. As a result, overexpression of *Dlx2* may affect different target genes in different stages. Third, maxillary cells at E10.5 and E12.5 may express different sets of transcriptional co-activators/co-repressors that function collaboratively with *Dlx2*, leading to different transcriptional outcomes.

By performing scRNA-Seq and comparing the pseudotime development trajectories of wildtype and *wnt1* ^*cre*^ *; Rosa26* ^*Dlx2/-*^ cells, we found that overexpression of *Dlx2* had little effect on the differentiation trajectory of cells and did not cause alterations in cell fates, or loss of specific cell types. Thus, although the overexpression of *Dlx2* resulted in abnormal gene expression in early maxillary processes, this did not significantly change the direction of cell development. Rather, the main effects of *Dlx2* overexpression are decreased cell proliferation and premature differentiation. The precocious differentiation was sufficient to disrupt the normal developmental timing of tissues, which resulted in defects of maxillary development and a series of other phenotypes, highlighting the intricacies of the gene regulation of craniofacial development.

As a transcription factor, there are many downstream target genes of *Dlx2* in this regulatory process. Our CUT&Tag results suggest *Dlx2* may regulate some genes in collaboration with Mnt and Runx2. However, more experimental evidence is needed to further confirm their co-occupancy at Dlx2 binding sites and collaboration in transcriptional regulation. In addition to the previously recognized Wnt signaling pathway, we found that Notch signaling pathway was also regulated by *Dlx2*. The Notch signaling pathway has a central role in cell fate specification and differentiation (Yun et al., 2002). Early activation of this pathway is a common feature of most potent inducers of neural differentiation (Teratani-Ota et al., 2016) and there was a direct link between the level of Notch activation, pro-osteogenic gene expression and corresponding osteogenic induction (Kostina et al., 2021).

Although this study is an in-depth analysis of the regulatory role and mechanism of *Dlx2* in the early stage of maxillary process development, the roles of approximately 300 direct regulatory gene sites in the downstream complex regulatory network are still unclear. A large number of *in vivo* or *in vitro* experiments are still needed to verify the targets. Still, our study provides important information and resources that will facilitate the functional dissection of the *Dlx2* regulatory network down the road.

## Data Availability

The data presented in the study have been deposited in the National Center for Biotechnology Information (NCBI) Gene Expression Omnibus (GEO) under the accession codes GSE217214. The bulk RNA-Seq data for E12.5 mouse maxillary process have been deposited in GEO under the accession code GSE185279. The scRNA-Seq data for E12.5 wildtype mice maxillary process have been deposited in GEO under the number GSE161143.

## Supplementary material

Supplementary Figure S1 The top 10 differentially expressed genes of each cell cluster in Figure 3B.

Supplementary Figure S2 The quantification of the pseudotime difference between wildtype and *wnt1* ^*cre*^ *; Rosa26* ^*Dlx2/-*^ cells.
