# Supplementary figures and images for: Effects of Dlx2 overexpression on the genes associated with the maxillary process in the early mouse embryo
Source: Front Genet. 2023 Feb 20;14:1085263. doi: 10.3389/fgene.2023.1085263 (PMC9986417; doi:10.3389/fgene.2023.1085263)

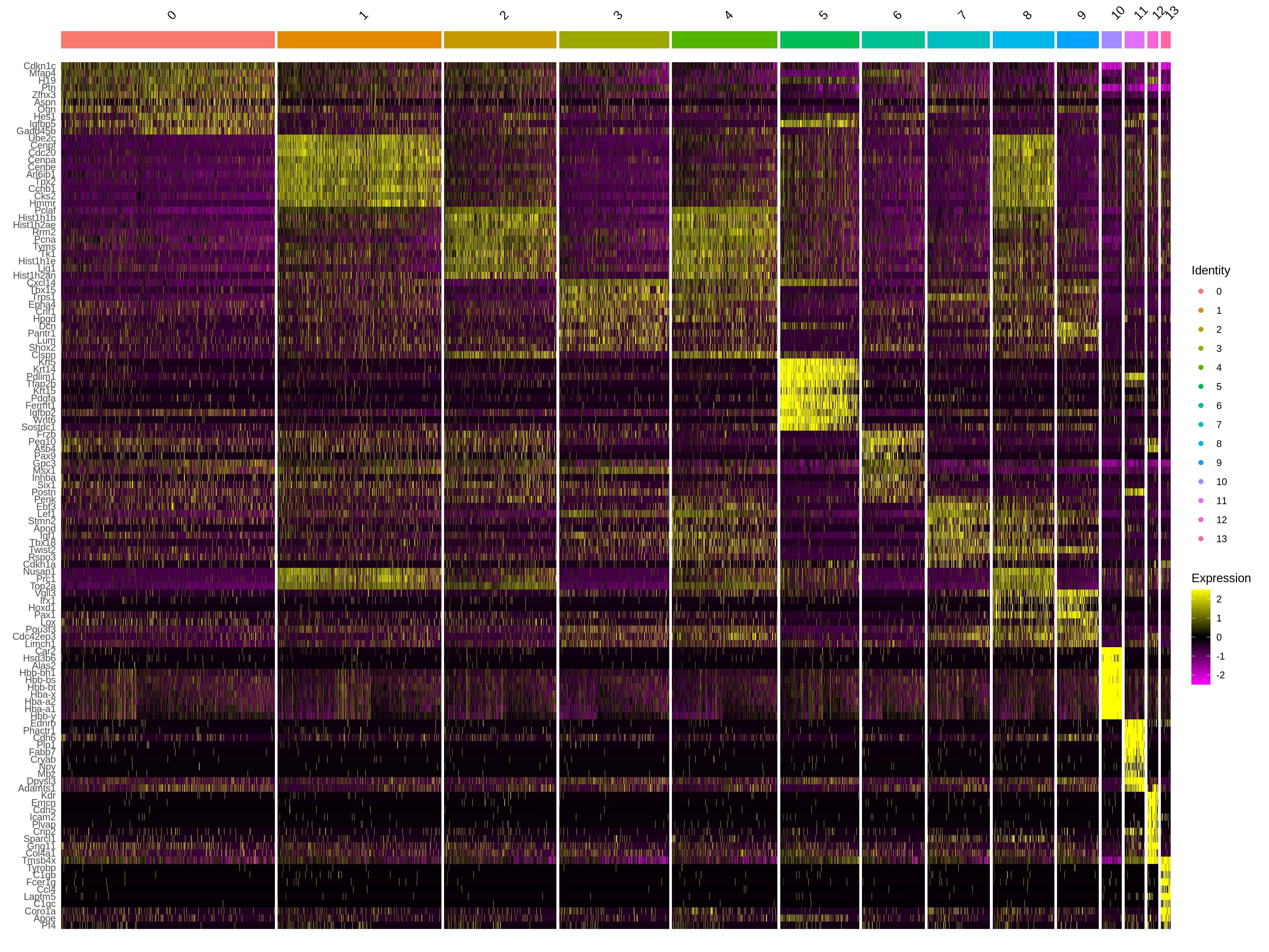

Supplement: Supplementary file 1 [file Image1.jpeg]

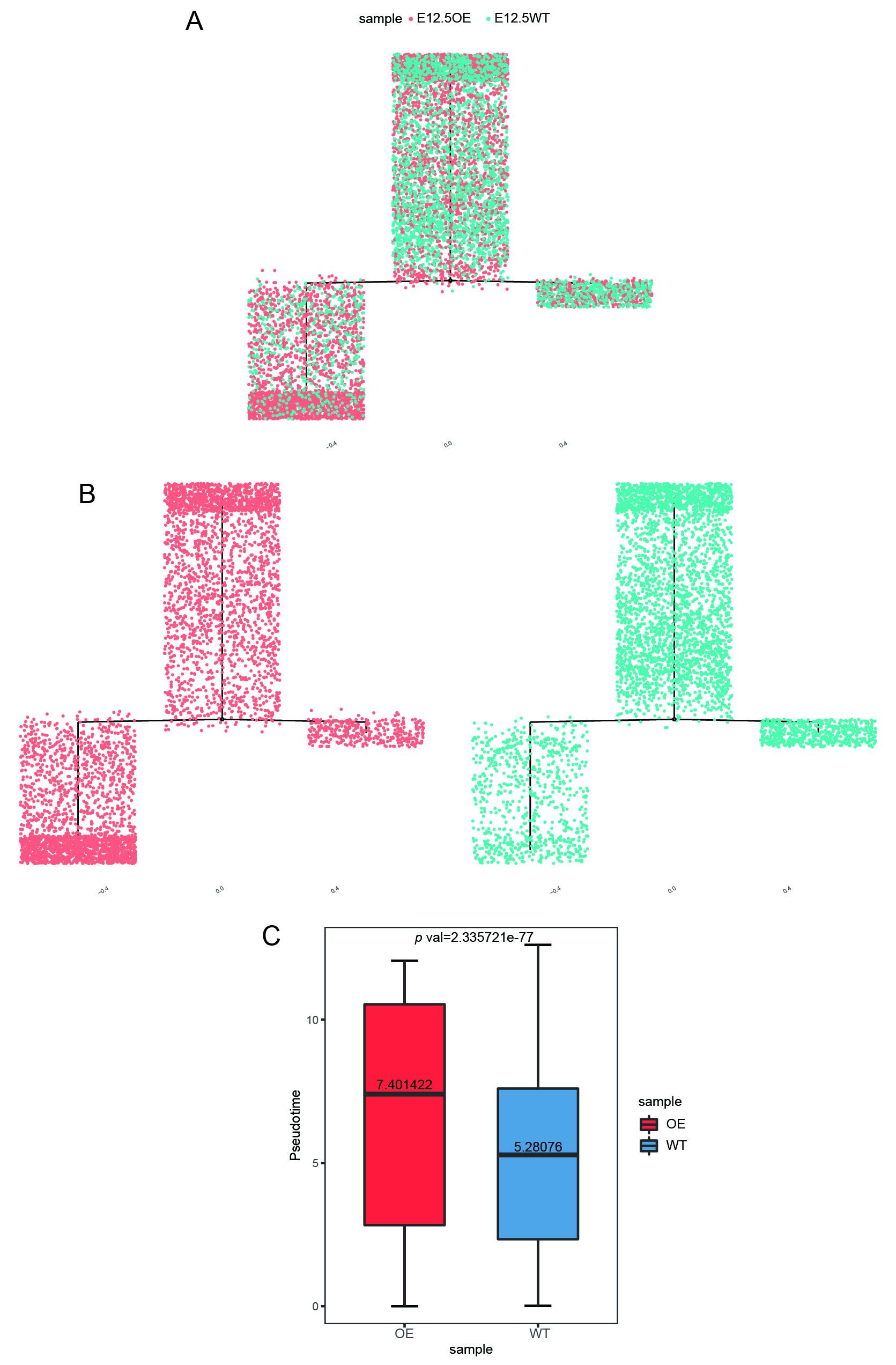

Supplement: Supplementary file 2 [file Image2.jpeg]
